# Newly Identified Regulators of Ovarian Folliculogenesis and Ovulation

**DOI:** 10.3390/ijms21124565

**Published:** 2020-06-26

**Authors:** Eran Gershon, Nava Dekel

**Affiliations:** 1 Department of Ruminant Science, Agricultural Research Organization, PO Box 6, Rishon LeZion 50250, Israel; eran.gershon1@mail.huji.ac.il; 2 Department of Biological Regulation, Weizmann Institute of Science, Rehovot 76100, Israel

**Keywords:** folliculogenesis, ovulation

## Abstract

Each follicle represents the basic functional unit of the ovary. From its very initial stage of development, the follicle consists of an oocyte surrounded by somatic cells. The oocyte grows and matures to become fertilizable and the somatic cells proliferate and differentiate into the major suppliers of steroid sex hormones as well as generators of other local regulators. The process by which a follicle forms, proceeds through several growing stages, develops to eventually release the mature oocyte, and turns into a corpus luteum (CL) is known as “folliculogenesis”. The task of this review is to define the different stages of folliculogenesis culminating at ovulation and CL formation, and to summarize the most recent information regarding the newly identified factors that regulate the specific stages of this highly intricated process. This information comprises of either novel regulators involved in ovarian biology, such as *Ube2i*, Phoenixin/GPR73, C1QTNF, and α-SNAP, or recently identified members of signaling pathways previously reported in this context, namely PKB/Akt, HIPPO, and Notch.

## 1. Folliculogenesis

Folliculogenesis is initiated during fetal life. The migration of the primordial germ cells (PGCs) to the embryonic genital ridge [1] may, in fact, be considered as the earliest event along this process. Once the PGCs localize at the genital ridge, the bipotential gonad is formed and further transforms into either an ovary or a testis. Simultaneous development of the somatic, supporting cells in the gonad [2] plays a crucial role in PGCs differentiation. Cell migration and differentiation, which are part of gonad formation, are subjected to regulation by multiple local as well as remote factors to be discussed in detail in this review. The information presented herein has been published during the past five years (2016 and on), thus not including regulators, such as FIGLA, Forkhead box L2 (FOXL2), and Transforming growth factor β (TGFβ) family members, KITL/KIT signaling, PI3K/Akt, Leukemia inhibitory factor (>LIF), Notch signaling, cAMP, cGMP, and their corresponding phosphodiesterases (PDEs), Epidermal growth factor receptors (EGFR) and ligands, etc. These regulators have been discussed in detail previously [3].

### 1.1. Primordial Follicle Formation and Their Activation

Differentiation of the female PGCs into oogonia, is followed by their proliferation. However, as cytokinesis in this case is incomplete, they form clusters (syncytia), also known as nests of germ cells, connected and synchronized by intercellular bridges. In the mouse, the individual oogonia within the clusters embark on meiosis from 13.5 days post coitum (dpc) to become oocytes [4]. The entire oocyte population proceeds through the first cycle of meiosis, arresting at diplotene on 18.5 dpc. Between 17.5 dpc to day 5 postpartum (dpp), germ cells nests begin to breakdown forming the primordial follicles. Each primordial follicle consists of an oocyte surrounded by one layer of flattened epithelium (pre-granulosa cells) that will differentiate to form the granulosa cells (GCs). Primordial follicle formation is accompanied by a massive cell lose [5]. Most of the primordial follicles remain quiescent and die at this dormant state. Those that survive serve as the ovarian follicle reserve, and are recruited to develop and establish the pool of growing follicles. A large fraction of these growing follicles is destined to go through another massive death wave, defined as oocyte degeneration, with only a minority activated to transform into primary follicles [6]. The transition into primary follicles is characterized by a morphological change of the GCs, from flattened to cuboidal cells [6].

### 1.2. From the Primary to the Preantral Follicle

The primary follicles develop into the secondary follicles, containing each a growing oocyte surrounded by two or more layers of proliferating GCs. These follicles grow further to reach the preantral stage. At this point a basement membrane is formed outside the GCs and a new layer of somatic cells, the theca, divided into the interna and externa compartments, encapsulates the follicles. The theca cells layer serves as the source of androgens, which are the substrates for estrogen production by the GCs [7]. Blood supply is limited to the theca cells and the vessels do not penetrate beyond the basement membrane. Under these conditions the inner follicle stays avascularized [8].

The growth of the follicles from the primordial to the preantral stage is independent of gonadotropin stimulation, but rather controlled by bidirectional communication of local signals, originating from both, the oocyte and the somatic cells. The somatic cells of the follicles support oocyte growth and development, while the oocyte plays a critical role in regulating proliferation and differentiation of both, the GCs and the theca cells [3,9,10]. Small molecules of up to 1Kd are transferred through gap junctions, established between the different follicle cell types including the oocyte [11], whereas proteins elicit their action via their corresponding receptors.

### 1.3. The Antral and the Graafian Follicle

Fluid filled cavities appearing within the preantral follicles are finally united to form the antrum. Antral follicles are characterized by two GCs subpopulations: the cumulus cells surrounding the oocyte, and the mural GC that line the follicle wall [9]. Most of the antral follicles will go through atretic degeneration [12], and only a few of them will respond to the pituitary gonadotropins follicle-stimulating hormone (FSH) and luteinizing hormone (LH) [13,14]. The first gonadotropin affecting the antral follicle, FSH, is responsible for GCs survival and their proliferation, estradiol production and LH receptors expression [9,15].

Antral follicles expressing a high concentration of LH receptors will respond to this gonadotropin to transform into the preovulatory/Graafian follicles [3]. The preovulatory LH surge activates the Graafian follicles, generating a sequence of events, including oocyte maturation, cumulus cell mucification, follicle rupture, and the subsequent extrusion of the oocyte. This series of events is collectively referred to as ovulation.

## 2. Novel Regulators of Fulliculogenesis

A large body of novel factors involved in regulation of folliculogenesis has been identified in the recent years. Those depicted herein (summarized in Table 1), together with the previously described regulators, constitute a highly complex mechanistic network, essential for successful reproduction.

### 2.1. Transcription Factor SP1

Transcription factor SP1, which regulates genes involved in cell proliferation and differentiation [16], is a member of the protein/Krüppel-like factor (Sp/KLF) transcription factors family [17]. This factor was recently localized in ovarian somatic cells at 16.5 dpc [18]. During follicle formation, at 18.5 dpc up to 3 dpp, SP1 protein levels increase in both, the oocytes and the somatic cells. Knocking down SP1 in ovaries of fetal mice, resulted in extensive apoptosis of oocytes as well as that of the cells in the ovarian surface epithelium, with the subsequent decline in the number of primordial follicles. The effects of SP1 were shown to be mediated by Notch2. These findings point at SP1 as a novel essential factor that functions mostly in somatic cells to promote development of the pregranulosa cells, essential for the formation of primordial follicles. The expression of Notch in pre-granulosa cells at 18.5 dpc, and its essential role in encapsulation of germ cell to form the primordial follicles, has been shown previously [19,20,21,22].

### 2.2. mTOR

mTOR is a downstream mediator of the PI3K/Akt pathway [23]. Previous studies demonstrated that the PI3K/Akt pathway mediates both, FSH and GDF9 survival effects in GCs [24,25,26]. A later study showed the indispensable role of Akt in early folliculogenesis using Akt null mice. In these mice breakdown of the germ cell cysts was defective and multiovular follicles were observed. The ovaries of these mice contained significantly fewer primary follicles [27]. Interestingly, oocyte-specific deletion of *Pten*, an Akt regulator, resulted in premature activation of the entire pool of primary follicles followed by their depletion in early adulthood [28]. Along this line, oocyte-specific deletion of *Pdk,1* another regulator of Akt, resulted in premature ovarian failure due to accelerated clearance of primary follicles from their dormant state [29]. In line with these variable functions of PI3K/Akt pathway in mouse ovarian follicles, the essential role of mTOR for the development, survival and activation of the follicle before ovulation has been recently reported [30,31]. Specific deletion of mTOR in the oocytes at the primordial stage severely reduced ovulation rate, further compromising the developmental competence of oocytes that did ovulate. The defective oocytes were characterized by either abnormal metaphase II (MII) spindle or incomplete cytokinesis. These oocyte defects were attributed to accumulation of double strand breaks leading to their loss with age. Interestingly, this oocyte-directed deletion lessened the number of secondary follicles, suggesting that mTOR is indispensable for follicular development beyond the primary stage. RNA-seq analysis of the mTOR depleted oocytes discovered differential expression of 979 transcripts. Gene-enrichment analysis revealed down-regulation of genes controlling key oogeneic processes, oocyte mRNA decay, epigenetic and transcriptional control, cell cycle, microtubule-related processes, oocyte–granulosa communication as well as those involved in the development and survival of oocytes companion GC [32,33,34,35,36,37]. Genes associated with mRNA decay that could dysregulate the transcript dosage of certain key factors for oocyte and follicle development were upregulated. These transcriptomic changes might explain the observed defects in oocyte and the failure of follicular development, observed in the mTOR-deleted oocytes at the primordial stage. Enigmatically, the transcriptome of mTOR-deleted growing oocytes revealed only 85 differentially expressed transcripts, whereas, proteomic analysis of these oocytes detected difference in the expression of 237 proteins [31].

### 2.3. The E2 SUMO-Conjugating Enzyme (Ube2i)

SUMOylation is the covalent attachment of a small ubiquitin-like modifier (SUMO) protein to a lysine residue in a target protein for degradation; its substrates are often transcription factors [38]. The covalent attachment of SUMO to substrate proteins occurs through an enzymatic cascade similar to that of ubiquitination [39]. Ubiquitin conjugating enzyme E2I (*Ube2i*), also known as UBC9, is the main enzyme in the SUMOylation cascade. Systemic deletion of *Ube2i* is embryonic lethal as a result of mitotic defects in chromatin condensation, segregation, and nuclear organization [40]. Inhibition of *Ube2i* in mouse meiotically arrested germinal vesicle (GV) oocytes in vitro disrupted meiotic maturation and caused defects in spindle organization [41]. Overexpression of *Ube2i* by injecting the corresponding mRNA to meiotically incompetent oocytes stimulated transcription [42]. Interestingly, during meiotic progression in *C. elegans*, SUMOylation is responsible for regulating localization of various proteins [43]. Mouse females with oocyte-targeted deletion of *Ube2i* were infertile, exhibiting reduced ovarian size and lower ovulation rate. The few oocytes that ovulated, underwent GV breakdown, but failed to extrude a polar body. Although females with *Ube2i*-deleted oocytes were infertile, their ovaries at the age of three weeks did contain primary and preantral follicles. However, ovaries recovered from eight-week-old such females, exhibited the absence of preantral and antral follicles as well as CL. These were accompanied by elevated serum FSH and reduced ovarian AMH levels. This phenotype became more sever with age. At 12–14 weeks of age, a significantly reduced number of primordial, secondary and antral follicles, along with an increased number of atretic follicles were detected, with a complete follicle depletion at the age of 6 months. RNA-seq analysis, comparing *Ube2i* oocyte-targeted deletion and wild type ovaries revealed 208 upregulated and 377 downregulated genes. Significant downregulation was detected in known oocyte-expressed factors, such as bone morphogenetic protein 15 (*Bmp15*), ret finger protein-like 4 (*Rfpl4*), spalt-like transcription factor 4 (*Sall4*), and SMAD family member 6 (*Smad6*). Significantly upregulated genes included the oocyte expressed synaptonemal complex protein 3 (*Sycp3*), natriuretic peptide receptor 1 (*Npr1*) and kelch-like family member 1 (*Klhl1*). In addition, deletion of *Ube2i* in the oocyte revealed that SUMOylation regulated NOBOX transcriptional activity. This study suggests that SUMOylation in the oocyte is indispensable for oocyte as well as the somatic cells development [44].

### 2.4. The Hippo Pathway

Activation of the Hippo pathway starts with a sequential phosphorylation of serine threonine kinase 3 (STK3) and STK4, (also known as MST1/2), triggering phosphorylation of the large tumor suppressor 1/2 (LATS1/2) kinases, that in turn phosphorylate the transcriptional co-activators, Yes-associated protein1 (YAP1) and WW domain-containing transcription regulator protein 1 (WWTR1, also known as TAZ). The phosphorylated YAP1 and WWTR1 are sequestered to the cytoplasm, and their action as transcriptional co-activators is prevented [45,46]. In the ovaries, YAP1 is localized in the cytoplasm of GC of primordial and secondary follicles [47,48]. Follicular development in the ovary is accompanied by a reduction in MST1 and LATS2 and elevation in YAP1 expression, accompanied by a decrease in MST1 phosphorylation, indicating its inactivation [47]. Interestingly, testosterone and estradiol stimulate GC proliferation and follicular growth by inducing YAP1 transportation into the nucleus and its activation [49]. It has also been reported that mechanical fragmentation of the ovary results in transport of YAP1 to the nuclei of GCs followed by stimulated expression of connective tissue (CCN) growth factors as well as Baculoviral inhibitors of apoptosis repeat containing (BIRC) apoptosis inhibitors, via the Akt pathway [50,51]. The effect of the Hippo pathway on primordial follicle activation has been demonstrated by YAP1 knockdown, which brought about a significant elevation in the number of primordial follicles accompanied by a decrease in the number of primary follicles. The reciprocal effect, manifested by a reduced number of primordial follicles and elevation in secondary follicles, is obtained upon YAP1 overexpression [47,52]. These findings are supported by another study, in which a specific deletion of YAP1 in GCs of primordial follicles resulted in subfertility which was subsequent to increased cell apoptosis, reduced number of CL and a decline in ovarian size [48]. Taken together these studies suggest that YAP1 mainly induces the transition of follicles from the primordial to the primary stage.

The other components of the Hippo pathway, such as, MST1/2, salvador (SAV)1, LATS1/2, and TAZ were also detected in the cytoplasm of GCs and theca cells, as well as oocytes of follicles at all sizes, with a lower levels in the CL [51]. A function of the Hippo pathway earlier in folliculogenesis is suggested in ovaries lacking LATS1; most oocytes in these ovaries were not surrounded by GCs resulting in a reduced number of primordial follicles. Furthermore, isolating LATS1 null ovaries from mice on the first dpp and their culture in vitro for seven days was accompanied by the presence of degenerative cyst-like structures containing cuboidal or squamous epithelium, possibly representing oocyte nests which fail to breakdown [53].

### 2.5. C1q/Tumor Necrosis Factor-Related Protein 3 (C1QTNF3)

C1q/tumor necrosis factor-related protein 3 (C1QTNF3) is a new member of the C1q/TNF-related protein family (CTRPs). These family members have been shown to be paralogs of adiponectin [54], the deletion of which led to elevated numbers of atretic follicles, impairing late folliculogenesis [55]. C1QTNF3 is mainly detected in GCs and oocytes of large follicles. In addition, C1QTNF3 mRNA expression level is upregulated by FSH and downregulated by LH as indicated by the higher C1QTNF3 levels observed in the GCs of the large follicles as compared to CL [56]. This study also found that C1QTNF3 induced proliferation of GCs in all follicles, from primary to antral stage by increasing the expression levels of CCND2. It was further revealed that C1QTNF3 protected GCs from apoptosis by reducing the activated CASP3 levels. In agreement, atretic follicles expressed reduced levels of C1QTNF3.

### 2.6. Neuropeptide Phoenixin and Its Receptor GPR173

Phoenixin-14 and phoenixin-20 are products of the precursor small integral membrane protein 20 (SMIM20) cleavage. The most abundant expression of phoenixins was found in the hypothalamus, with a lesser expression in the ovary [57]. Phoenixin binding to the orphan receptor G-Protein coupled receptor 173 (GPR173) [58,59] induced GnRH receptor expression leading to LH release from pituitary cultures [57]. In addition, intracerebroventricular injection of phoenixin increased GnRH and LH plasma levels [58,60].

In the human and mouse ovary, phoenixin and GPR173 are localized in the GCs and oocytes at all stages of folliculogenesis, from primary to the Graafian follicle. In the primary follicles GCs expressed low levels of phoenixin and GPR173 and their expression was increased as the follicle developed, with the highest levels found in the antral follicle and the CL [61]. In agreement, ovarian tissue slices cultured with phoenixin exhibited a dose-dependent follicular growth [62,63]. Furthermore, These ovarian tissue slices contained a significantly higher proportion of secondary and antral follicles, but a lower proportion of primordial follicles [61], showing an increased estrogen production. Furthermore, the addition of phoenixin to the culture medium markedly increased the number of mature MII oocytes and lowered the percentage of meiotically arrested GV oocytes. Supporting these results, incubation of the HGrC1 human GC line, in the presence of phoenixin significantly increased cell proliferation and the expression of GPR173, cAMP responsive element binding protein 1(CREB1), CYP19A, FSHR, LHR, and KITL but decreased the mRNA levels of NPPC. Finally, phoenixin also induced estrogen production in cultured HGrC1 cells [61].

### 2.7. Soluble N-ethylmaleimide-Sensitive Factor (NSF) Attachment Protein (α-SNAP)

Soluble N-ethylmaleimide-sensitive factor (NSF) attachment protein (α-SNAP) together with the ATPase NSF, dissociates the inactive cis-SNAP receptors (SNARE) complexes, preparing them for additional rounds of membrane fusion events [64]. Alternately, α-SNAP regulates, in a NSF-independent fashion, cellular processes including cell-cell and cell-extracellular matrix adhesion, autophagy, apoptosis and others [65]. A recent study found that α-SNAP is expressed in the GCs and that its expression is upregulated by human chorionic gonadotropin (hCG), an LH analog that induces ovulation. This study also showed that α-SNAP expression was higher in ovaries of 60-day-old mice as compared to ovaries of 30-day-old mice, indicating that α-SNAP may be involved in follicle development and maturation of GCs [66]. Supporting this assumption, ovaries isolated from mice carrying a missense mutation at amino acid residue 105 for an isoleucine (M105I) in the *Napa* gene, thus expressing reduced α-SNAP levels, hyh mice, [67,68], are smaller with a significant increase in atresia and a higher rate of apoptotic signs, resulting in a reduced total number of follicles [66]. In correlation with this finding, a significantly reduced numbers of preantral, early antral and antral follicles after hCG administration was observed. The reduced follicular development and the increased incidence of atresia and apoptosis in the hyh mice ovaries resulted in a lower ovulation rate and a decline in reproductive efficiency of the α-SNAP-mutant females. This study suggests that α-SNAP plays a critical role in folliculogenesis by balancing follicular atresia [66].

### 2.8. Immune Cells

Macrophages (MΦs) are immune cells shown to play diverse roles in ovarian events, including follicular growth [69]. There are two types of MΦs, M1 MΦs exhibiting inflammatory effects and M2 MΦs that are anti-inflammatory and possess remodeling effects [70]. A recent study demonstrated that folliculogenesis was not impaired upon depletion of M2 MΦs [71]. Nevertheless, depletion of M1-like MΦs and dendritic cells (DCs) resulted in follicular atresia, associated with hemorrhages. In addition, the proportion of antral follicles onward was severely decreased. Furthermore, in the absence of M1-like MΦs cells and CD11c+ dendritic cells (DCs), the angiogenic factor platelet derived growth factor subunit B (PDGF-B) signal was negative in stromal lesions. Furthermore, mature blood vessels around the follicles, identified by CD34+ vascular endothelial cells and PDGF-Rβ+ pericytes, were decreased. On the other hand, the angiogenic factors vascular endothelial growth factor (VEGF) and matrix metallopeptidases-9 (MMP-9)/ metallopeptidase inhibitor-1 (TIMP-1), derived from M2 MΦs, were not affected. This study suggests that M1 MΦs but not M2 MΦs are crucial for folliculogenesis. However, since in CD11c DTR mouse model, both, M1-like MΦs and DCs are depleted [72], the role of DCs during folliculogenesis cannot be excluded.

### 2.9. The Ovarian Fat Pad-Secreted Factors

Studies on the adipose tissue around lymph nodes, [73] next to blood vessels and in the pericardium [74,75] revealed its paracrine effect. Along this line, more recent studies demonstrated that removal of mouse ovarian fat pad resulted in a high proportion of follicles blocked at the secondary stage, with less follicles developed to early antral and Graafian follicles. Subsequently, fewer follicles ovulated and thus fewer CL were formed, with only a small fraction of the ovulated oocytes reaching MII. The lower fraction of antral and Graafian follicles corresponded with a decrease in the expression levels of ovarian steroidogenic enzymes such as aromatase (CYP19), cytochrome P450 side chain cleavage (CYP11A1), 17α-hydroxylase/17, 20-lyase (CYP17) and 3β-hydroxysteroid dehydrogenase (3β-HSD), accompanied by reduced levels of estrogen and FSH but increased levels of LH. In addition, the level of FSH receptor mRNA, decreased significantly with a significant increase in the levels of the LH receptor mRNA. The endocrine maladies observed led to estrous cycle disorders [76,77].

The removal of the ovarian fat pad also increased the number of atretic follicles along with a decreased abundance of noncleaved apoptosis related proteins CASP3, CASP8 and CASP9 and an increase in the cleaved CASP3 expression, accompanied by elevation in cleaved CASP9 levels. Removal of ovarian fat pad also reduced the mRNA and protein levels of adiponectin, and lipocalin-2 (*Lcn2*). The protein levels of VEGF were also diminished significantly. A tendency of decreased leptin, hepatocyte growth factor (HGF), and C1QTNF3 was also observed. Finally, the removal of the ovarian fat pad significantly decreased the levels of phosphorylated Akt, the ratio of phosphorylated Akt to total Akt and the abundance of non-phosphorylated YAP1, increasing the phosphorylation levels of YAP1. In agreement with Akt inactivation, a significant decrease in mTOR phosphorylation and the ratio of phosphorylated mTOR to total mTOR were observed in the ovarian fat pad-deficient ovaries. Both, PTEN and mTOR are related to Akt phosphorylation and activation [77].

Taken together, these findings suggest that the fat surrounding the ovary secrets factors that may play a paracrine role essential for folliculogenesis. Nevertheless, further studies are required to identify these factors and reveal their specific functions in order to define the relationship between the fat tissue adjacent to the ovary and follicle development and function.

## 3. Ovulation

As mentioned above, the preovulatory, pituitary LH surge, activates a multistep process in the ovulatory follicles stimulating a sequence of events, collectively defined as ovulation. The ovulatory response to LH consists of resumption of meiosis in the oocyte, mucification and expansion of the cumulus and rupture of the follicle culminating in the release of the cumulus–oocyte complex (COC). Once the COC is released, the residual GCs and thecal cells differentiate into the CL in a process defined as luteinization.

### 3.1. Resumption of Meiosis

In response to the LH surge, the gap junctions, which connect the oocytes and the surrounding cumulus cells close [78], thus preventing the transfer of both, cAMP and cGMP to the oocyte. The termination of cAMP supply, together with its increased hydrolytic degradation by PDE3A, achieved in the absence of cGMP, lead to reduction in intra-oocyte cAMP levels [79], and the subsequent PKA inactivation. Upon the release of the PKA inhibition, Cdc25B dephosphorylates and activates CDK1, switching on the resumption of meiosis [80,81]. Meiosis in the follicular oocyte progresses through the first metaphase, anaphase and telophase. Upon completion of the first round of meiosis the homologous chromosomes separate and the first polar body is extruded. Without and intervening interphase the oocytes enters the second metaphase and arrests again. Oocytes released from the follicle at ovulation have not completed the second round of meiosis. This event, including sister chromatid separation, occurs in the oviduct, upon fertilization, in response to sperm penetration [82].

### 3.2. Cumulus Mucification

Mucification of the cumulus, also known as cumulus expansion, is characterized by hyaluronan-rich extracellular matrix secretion and its apposition in the cumulus-oocyte complex [83]. The production of the structural backbone of the COC extracellular matrix, hyaluronan, is catalyzed by the hyaluronan synthase 2 (HAS2), the expression of which is upregulated by LH. Another enzyme crucial for cumulus mucification is prostaglandin synthase 2 (PTGS2, also known as COX2), the rate-limiting enzyme in the synthesis of prostaglandins [9]. A previous study demonstrated that deletion of COX2 in mice led to anovulation, leaving the oocytes trapped within the CL [84].

### 3.3. Luteinization and CL Formation

Ovulation culminates in follicle rupture and the release of a fertilizable ovum from the follicle to the oviduct. This event is followed by differentiation of the remaining theca and GCs into luteinizing cells of the CL. The role of the CL is to produce and secret progesterone, a crucial hormone for pregnancy establishment and maintenance. The formation of the CL involves neovascularization and endothelial cell migration, thus forming a characteristic dense blood capillary network. Up to ovulation the follicle vascularization is limited to the theca cell compartment. These blood capillaries formed upon ovulation penetrate the CL that turns highly vascularized, allowing efficient cholesterol, hormones, and nutrients supply to the luteal cells as well as effective secretion of progesterone. VEGF and angiopoietins that promote endothelial cell migration and proliferation are the major regulators of CL vascularization [85,86].

### 3.4. Novel Regulators of Ovulation

The large number of novel factors identified recently as regulators of ovulation clearly contribute to our understanding of the mechanistic complexity of this event (summarized in Table 2). Among those genes, differentially expressed in porcine preovulatory follicles (POFs), cAMP-dependent protein kinase, histone binding protein RBP4, reticulocalbin, vimentin, and calumenin were included [87]. The genes elevated in the newly formed CL included albumin, farnesyl diphosphate synthase, serine protease inhibitors, elongation factor-1, glutaredoxin, and selenium-binding protein. Modification in genes involved in cholesterol biosynthesis, cell death and survival and acute phase response, was also associated with luteal cells formation. In addition, in the newly formed CL differences in the abundance of proteins that included insulin growth factor-1, sterol regulatory element-binding transcription factor-1, and nuclear factor erythroid-derived 2 were identified. Taken together, this study identified novel proteins related to differentiation of POFs cells into the CL, mechanisms involved in luteal cell survival and pathways regulating steroidogenesis in the newly formed CL [87]. It is of course obvious that VEGF family members and their receptors are highly expressed in the ovulatory follicles [88,89].

#### 3.4.1. VEGFA, VEGFC, VEGFD, PGF and Their Receptors

As mentioned above VEGF family members and their receptors are key regulators of angiogenesis. Within this group, VEGFA is considered as the major stimulator of blood vessels formation, whereas VEGFC and VEGFD promote mainly lymph angiogenesis [90]. Nevertheless, blood vessels formation by the last two family members has also been reported [91]. Another VEGF family member is placental growth factor (PGF, [92]), mainly known for its role in embryonic and placental vessel formation [93].

PGF and VEGFA are expressed in both theca and GCs of the ovulatory follicle in cynomolgus monkey and their mRNA levels were elevated in isolated GCs in response to hCG, with the corresponding proteins found in the follicular fluids [91,94]. The injection of PGF antibody into the cynomolgus monkey ovulatory follicle resulted in abnormal rapture of the follicles with 50% of the injected follicles containing a trapped oocyte. These follicles exhibited compromised luteinization, characterized by a thinner granulosa layer as well as a reduced endothelial cells invasion and their very limited branching [94]. The injection of VEGFA into cynomolgus monkey ovulatory follicle also resulted in a failure of the follicle to rapture, displaying a thin apex and a trapped oocyte. Those follicles also showed impaired luteinization with a thin layer of GCs, almost no invasion of endothelial cells, and a complete absence of branching [91].

#### 3.4.2. Secretogranin II 

Secretogranin II (SCG2), a member of the chromogranin family of acidic secretory proteins plays a role in peptide hormones sorting and packaging into secretory vesicles [95]. It is expressed in a number of endocrine, neuroendocrine, and neuronal tissues, including the anterior pituitary gonadotrops [96]. Almost 90% of SCG2 is cleaved, generating three bioactive peptides, secretoneurin (SN), EM66, and manserin [96,97]. Although SN has been shown to be involved in many processes, including LH secretion and angiogenesis [97], the role of the other peptides is not fully understood as yet [98]. A recent study found that hCG induced upregulation in SCG2 mRNA levels in human, macaque monkey and rodents ovaries [99]. This study further showed that the SCG2 protein is localized in the theca, stroma and GCs in human and mice follicles and that its expression, in both species, is stimulated by hCG further demonstrating that SN significantly increased human ovarian microvascular endothelial cell migration. Using the endothelial sprouting assay, this study found that SN elevates the number of endothelial sprouts.

#### 3.4.3. The Hippo Pathway

As mentioned previously, YAP1 is involved in ovarian follicles development [47,48,52]. Consistent with oocyte-induced YAP1 activation in cultured GCs, YAP1 is localized to both, the nucleus and the cytoplasm, whereas, in co-cultures of GCs and oocytes YAP1 is mainly nuclear [100]. In addition, it has been shown recently that removing the oocyte from COCs resulted in elevated expression levels of Lats1 and Lats2, kinases that phosphorylate YAP1, which was reversible upon the addition of oocytes to the culture. Moreover, inhibition of SMAD2/3 in COCs also induced Lats2 kinase expression. Furthermore, verteporfin (VP), a YAP-TEAD inhibitor, in GCs culture, not only decreased cell number, but completely blocked the ability of oocytes to stimulate cell proliferation. In addition, in the absence of any other ovulatory signals, VP stimulated the expression of transcripts involved in cumulus mucification (Has2, Ptgs2, Ptx3, Tnfαip6). The expression of major steroidogenic transcripts including Star and Cyp11a1 was also increased significantly by VP, resulting in elevated progesterone secretion [100].

An acute exposure of COCs to EGF resulted in higher expression levels of the Hippo family members Stk3/4, Lats1, Lats2 and Wwtr1 as well as a significant increase in pYAP. However, a prolonged exposure to EGF, both in vitro and in vivo, significantly reduced the amount of total and phosphorylated LATS1 and significantly decreased total YAP1 expression. In contrast, WWTR1 was increased by a prolonged treatment with EGF. Furthermore, incubation of GCs in vitro in the presence of forskolin, which is an adenylate cyclase activator, led to translocation of YAP1 from the nucleus and its inactivation [49]. Administration of hCG to female mice increased the levels of phospho-MST1/2, which is a negative regulator of YAP1 [49]. Taken together, these results suggest that the LH surge via the EGF pathway triggers the Hippo pathway activation leading to YAP1 degradation and the subsequent cumulus cells differentiation and mucification.

#### 3.4.4. Chemerin and GPR1

Chemerin, also known as *TIG2* or RARRES2 [101], a recently discovered adipose cytokine, regulates fat formation and adipocyte metabolism [102]. Chemerin is secreted from white adipocytes and widely expressed in multiple tissues in the human body including the placenta and ovary [103]. Among the many functions of chemerin, it plays a role in inflammation and immune responses [104]. Chemerin binds to three receptors, chemokine like receptor 1 (CMKLR1), G protein-coupled receptor1 (GPR1) and chemokine (C-C motif) receptor like 2 (CCRL2) [105]. Although the effects of CMKLR1 and CCRL2-mediated chemerin, are known [106], no function has been reported upon its binding to GPR1.

Chemerin and its receptors are expressed in mouse, human and rat ovary and inhibit hormones secretion [107,108]. Incubating rat GCs with chemerin inhibited progesterone and estradiol production, as well as the insulin-like growth factor-induced, and the FSH-induced progesterone and estradiol secretion [109]. Another study demonstrated that chemerin inhibited hCG-induced key steroidogenic factors expression, including Star, P450scc, and 3β-Hsd with a subsequent reduction in progesterone secretion in mouse follicles and luteal cells [110]. These negative effects of chemerin on hCG-induced steroidogenic factors expression and progesterone secretion can be restored by either a PI3K inhibitor or a GPR1 antibody, suggesting that chemerin inhibition on hCG-induced progesterone secretion is mediated via GPR1 and PI3K pathway. This study further reports that GPR1 levels decrease throughout luteolysis and that blocking GPR1 activity in the CL, reduced caspase-3 mRNA levels, lowered the number of apoptotic luteal cells, and elevated progesterone serum level. These last results suggest that the chemerin/GPR1 signaling plays a role in promoting CL luteolysis.

#### 3.4.5. Thrombospondin 1(THBS1)

Thrombospondins are a family of vascular regulators that can act as either pro-angiogenic or anti-angiogenic factors [111]. Binding of thrombospondin 1 (THBS1) to its CD36 receptor, elicits an anti-angiogenic effect [112]. However, binding of THBS1 to the LRP1 receptors lead to a pro-angiogenic activity [113]. In the ovary, THBS1 and THBS2 are expressed in growing follicles, possibly playing a role in follicle development and follicle selection [114,115]. Their expression decreased as the follicle increase in size, with low levels in antral and large preovulatory follicles [114,116,117]. The increase in of THBS1 and THBS2 was observed in GCs and theca cells after the LH surge [117]. In bovine, vascular endothelial cells and vascular smooth muscle of the young CL expressed high levels of THBS1 and THBS2 [116]. A recent study demonstrated that THBS1 was abundant in the GCs of macaque monkey ovary. THBS4 was detected, but in low levels and THBS2 was barley expressed. In these cells hCG upregulated THBS1 and THBS4 levels with no effect on THBS2 expression [118]. THBS1 increased migration of cells and the number and length of capillary-like endothelial cell sprouts formed in culture of endothelial cells obtained from monkey ovulatory follicles in vitro. Ovulatory follicles injected with anti-THBS1 antibody in vivo failed to ovulate, as most oocytes were trapped in the follicles, with half of them containing a GV. In addition, THBS1 antibody-injected follicles exhibited a significantly thinner GC layer consisting of cells that displayed abnormal morphology. In addition, THBS1 reduced angiogenesis in the ovulatory follicles, and in THBS1 antibody-injected follicles, endothelial cells were located mainly near the basement membrane, exhibiting low penetration of the GC layer with no connection to stromal vessels. Moreover, THBS1 inhibition reduced the branching of capillary-like structures, accompanied by absence of red blood cells in the capillary luminal spaces. Red blood cells were detected in the antrum of THBS1 antibody-injected follicles.

#### 3.4.6. Vasorin

Vasorin (Vasn) is a newly identified negative regulator of TGFβ signaling [119], whose possible involvement in ovarian physiology has recently been studied [120]. This study demonstrated that Vasn was expressed by the GCs, and that its expression was upregulated by LH. Using a conditional knockout (cKO) mouse model, in which Vasn has been deleted specifically in the GCs of follicles from the secondary stage onwards, this study showed that, upon hormonal stimulation, ovulation size is doubled. The enhanced ovulatory response in the cKO mice was associated with overactivation of the TGF-β signaling pathway and a lower number of atretic antral follicles. The findings of this study identified Vasn as a novel regulator of the size of ovulation suggesting that it elicits its action via the TGFβ signaling pathway.

## 4. Implications to Human Infertility Treatments

Most of the factors mentioned herein were studied in human and/or primates (Table 1 and Table 2). Furthermore, a role of downstream mediators as well as upstream effectors of these factors, originally identified in animal models, was confirmed in human ovarian physiology. For example, Notch signaling, the downstream mediator of SP1, was shown to be expressed in human prenatal ovaries [121]. It has been further demonstrated that alterations in this gene may lead to polycystic ovarian syndrome (PCOS), poor response to infertility treatments and ovarian cancer [122]. Another example is the HIPPO signaling mentioned here as a regulator of both, folliculogenesis and ovulation. Major factors from this family were shown to be expressed in different stages of human folliculogenesis, suggesting a similar role to that demonstrated in mice [51]. In addition, PI3k/Akt signaling, an upstream effector of mTOR was demonstrated to activate primordial follicles up to the preovulatory stage with the development of maturation competent oocytes [123]. Further research in human ovaries should be performed in order to confirm the large body of accumulated findings in animal models. The generation of such information will potentially propose some novel strategies for infertility treatments.

## 5. Conclusions

The ovary is an extremely dynamic organ, undergoing remarkable changes from fetal life, throughout sexual maturation and up to menopause. During fertile life, the ovary changes periodically at each reproductive cycle, while preforming diverse functions. These include housing the reservoir of oocytes in dormant primordial follicles as well as continuously producing growing follicles and selecting a few of them to ovulate and release a fertilizable oocyte. In addition, the ovary is an endocrine gland and the main source for female sex hormones. As designated in this review, for the successful accomplishment of those functions, an accurate orchestration of numerous factors and their strict regulation is absolutely essential.

## Figures and Tables

**Table 1:** Newly identified regulators of folliculogenesis.

Gene	Function	Species
SP1	Development of pregranulosa cells. Formation of primordial follicles.	mouse
mTOR	Control of ovulation size. Acquisition of oocyte developmental competence. Development of secondary follicles.	mouse
*Ube2i*	Ovarian size. Control of ovulation rate. Faithful meiosis. Development of preantral & antral follicles. CL formation. AMH production. Protection of follicles from atresia.	mouse
YAP1	Transition of follicles from the primordial to the primary stage. Proliferation of granulosa cells. Protection of granulosa cells from apoptosis.	Mouse/human
C1QTNF3	Proliferation of granulosa cells Protection of granulosa cells from apoptosis	Human/mouse
Phoenixin/GPR173	Transition of primary follicles to secondary and their development to the antral stage. Stimulation of estrogen production Resumption of meiosis.	Human/mouse
Ovarian fat pad factors	Further development of secondary follicles. Protection from atresia. Resumption of meiosis. Production of estrogen. Expression of FSH receptor mRNA. Expression of LH receptor mRNA. Determination of the length of the estrous cycle	mouse
α-SNAP	Ovarian size. Protection from apoptosis. Development of preantral, early antral and antral follicles.	mouse
CD11c+ cells, M1 MΦs or DCs	M2 MΦs: regulation of folliculogenesis M1-like MΦs and DCs: Protection from atresia. Control of vessel permeability. Development of antral follicles.	mouse

**Table 2:** Newly identified regulators of ovulation.

Gene	Function	Species
VEGFA, VEGFC and D	Ovarian endothelial cell migration and their proliferation. Endothelial cell invasion into the granulosa layer and capillary branching. Rapture of the follicle and release of the oocyte. Luteinization.	Cynomolgus monkeys
SCG2/SN	Migration of the microvascular endothelial cells. Sprouting of blood vessels.	Human, monkey and rodents
Hippo pathway/YAP1	Cumulus cells differentiation and their mucification.	Mice
Chemerin and GPR1	Progesterone and estradiol secretion. Luteolysis of the corpus luteum.	Rat/mouse
THBS1	Granulosa cell proliferation. Reinitiation of meiosis. Ovulation. Endothelial cell migration. Stimulation of angiogenesis and branching of capillary-like structures.	Macaque monkey
Vasorin	Negative control of the size of ovulation.	mouse
